# Three dimensional fusion of electromechanical mapping and magnetic resonance imaging for real-time navigation of intramyocardial cell injections in a porcine model of chronic myocardial infarction

**DOI:** 10.1007/s10554-016-0852-x

**Published:** 2016-02-16

**Authors:** F. J. van Slochteren, R. van Es, M. Gyöngyösi, T. I. G. van der Spoel, S. Koudstaal, T. Leiner, P. A. Doevendans, S. A. J. Chamuleau

**Affiliations:** Department of Cardiology, University Medical Center Utrecht, E03.511, P.O. Box 85500, 3508 GA Utrecht, The Netherlands; Department of Cardiology, Medical University of Vienna, Vienna, Austria; Department of Radiology, University Medical Center Utrecht, Utrecht, The Netherlands; Interuniversity Cardiology Institute of the Netherlands (ICIN), Utrecht, The Netherlands

**Keywords:** Cardiac regenerative therapy, Intramyocardial stem cell injections, Electromechanical mapping, Myocardial fibrosis, MRI, Late gadolinium enhancement

## Abstract

For cardiac regenerative therapy intramyocardial catheter guided cell transplantations are targeted to the infarct border zone (IBZ) i.e. the closest region of viable myocardium in the vicinity of the infarct area. For optimal therapeutic effect this area should be accurately identified. However late gadolinium enhanced magnetic resonance imaging (LGE-MRI) is the gold standard technique to determine the infarct size and location, electromechanical mapping (EMM) is used to guide percutaneous intramyocardial injections to the IBZ. Since EMM has a low spatial resolution, we aim to develop a practical and accurate technique to fuse EMM with LGE-MRI to guide intramyocardial injections. LGE-MRI and EMM were obtained in 17 pigs with chronic myocardial infarction created by balloon occlusion of LCX and LAD coronary arteries. LGE-MRI and EMM datasets were registered using our in-house developed 3D CartBox image registration software toolbox to assess: (1) the feasibility of the 3D CartBox toolbox, (2) the EMM values measured in the areas with a distinct infarct transmurality (IT), and (3) the highest sensitivity and specificity of the EMM to assess IT and define the IBZ. Registration of LGE-MRI and EMM resulted in a mean error of 3.01 ± 1.94 mm between the LGE-MRI mesh and EMM points. The highest sensitivity and specificity were found for UV <9.4 mV and bipolar voltage <1.2 mV to respectively identify IT of ≥5 and ≥97.5 %. The 3D CartBox image registration toolbox enables registration of EMM data on pre-acquired MRI during the EMM guided procedure and allows physicians to easily guide injections to the most optimal injection location for cardiac regenerative therapy and harness the full therapeutic effect of the therapy.

## Introduction

Cardiac regenerative therapy for ischemic heart disease (IHD) targets local cardiac protection and regeneration by means of vasculogenesis, cardiomyogenesis, and matrix support [1]. Previous studies have shown that injection of stem/progenitor cells into the border zone of the infarcted area, stimulates cardiac repair via cell-to-cell contact and secretion of paracrine factors [2–5]. Therapeutic effects may importantly rely on the delivery and retention of the regenerative therapeutics on a location where oxygen and nutrients are available to enable survival. Hence, accurate identification of viable tissue in proximity of the myocardial infarct (MI) the infarct border zone (IBZ) is therefore of great importance. The gold standard technique to assess infarct size and location is late gadolinium enhancement (LGE) MRI.

Currently percutaneous intramyocardial cell injections are performed using the NOGA^®^XP electromechanical mapping (EMM) technique to identify the location of the infarction and the surrounding viable area. This technique measures local unipolar (UV) and bipolar (BV) electrical depolarization potentials and relative catheter tip displacements (Linear Local Shortening, LLS), to assess the local electrical and mechanical tissue characteristics and guide minimal invasive intramyocardial injections. A 3D magnetic tracking technique is used to create a three-dimensional interpolated reconstruction of the LV endocardium [6] and perform measurements and injections at multiple locations on the endocardium. Prior studies have investigated the relations between myocardial viability/perfusion/infarction and NOGA^®^XP parameters [7–9] by comparing bullseye representations of the parameters. Other studies in the field of electrophysiology have shown beneficial effects of the integration of electroanatomical mapping (EAM) and LGE-MRI for location of foci of ventricular arrhythmia enabling accurate targeting of ablations [10–12]. Different from the ablations, the injection locations in the context of cardiac regenerative therapy have no inherent reference (e.g. cessation of arrhythmia) of the correct location for the therapy. Since the infarcted myocardium is highly susceptibility for arrhythmias upon catheter contact, and the mapping procedure is time-consuming, EMM measurements do not completely cover the endocardium and data is interpolated in regions where no measurements are acquired. Moreover, in prior studies to assess the relation between viability/perfusion/infarction and NOGA^®^XP parameters, different definitions of these parameters are used, and consequently in literature a large variance of threshold values of the NOGA^®^XP parameters to accurately target IBZ can be found [7].

Since the non-transmurally infarcted border zone of the infarcted area is believed to be the preferred delivery site of the regenerative therapeutics [2], it is crucial to be optimally defined during the injection procedure. We have developed the 3D CartBox image integration toolbox to fuse NOGA^®^XP EMM and MRI data in a real time fashion [13]. With this technique we can combine the gold standard infarct imaging technique and local measures of myocardial electrical activity in order to more specifically determine the areas that are most suitable for cell injections. In this study we perform a retrospective in depth 3D multimodality analysis of the myocardial infarction areas with different infarct transmuralities in a porcine model of chronic myocardial infarction using 3D CartBox. Thereby we aim to evaluate the performance of the NOGA system to identify regions with a specific infarct transmurality (IT) and wall thickening (WT) as measured using MRI.

## Methods

### Animals

For this study 2 animal models were used. In model 1 (LCX) myocardial infarction (MI) was induced by 75 min of proximal left circumflex coronary artery (LCX) balloon occlusion as previously described [14]. Three 6-month old female Dalland Landrace pigs (60–70 kg; IDDLO, Lelystad, The Netherlands) were pre-treated with clopidogrel 75 mg/day for 3 days and amiodarone 400 mg/day for 10 days. Experiments in Utrecht were performed accordance with the “Guide for the Care and Use of Laboratory Pigs” prepared by the Institute of Laboratory Animal Resources and with prior approval by the Animal Experimentation Committee of the Faculty of Medicine, Utrecht University, The Netherlands. In model 2 (LAD) MI was induced in 14 3-months old domestic pigs by 90 min of percutaneous balloon occlusion of the left anterior descending coronary artery (LAD) after the second diagonal branch, followed by reperfusion. Pigs were pre-treated with clopidogrel (300 mg) and aspirin (250 mg) 1 day before procedure, and treated with daily dose of 75 mg clopidogrel and 100 mg aspirin. The experiments were performed in accordance with the “Guide for the Care and Use of Laboratory Pigs” prepared by the Institute of Laboratory Animal Resources and with prior approval by The Experimental Animal Care and Use Committee at the Faculty of Animal Science of the University of Kaposvar (Hungary).

**Fig. 1:**
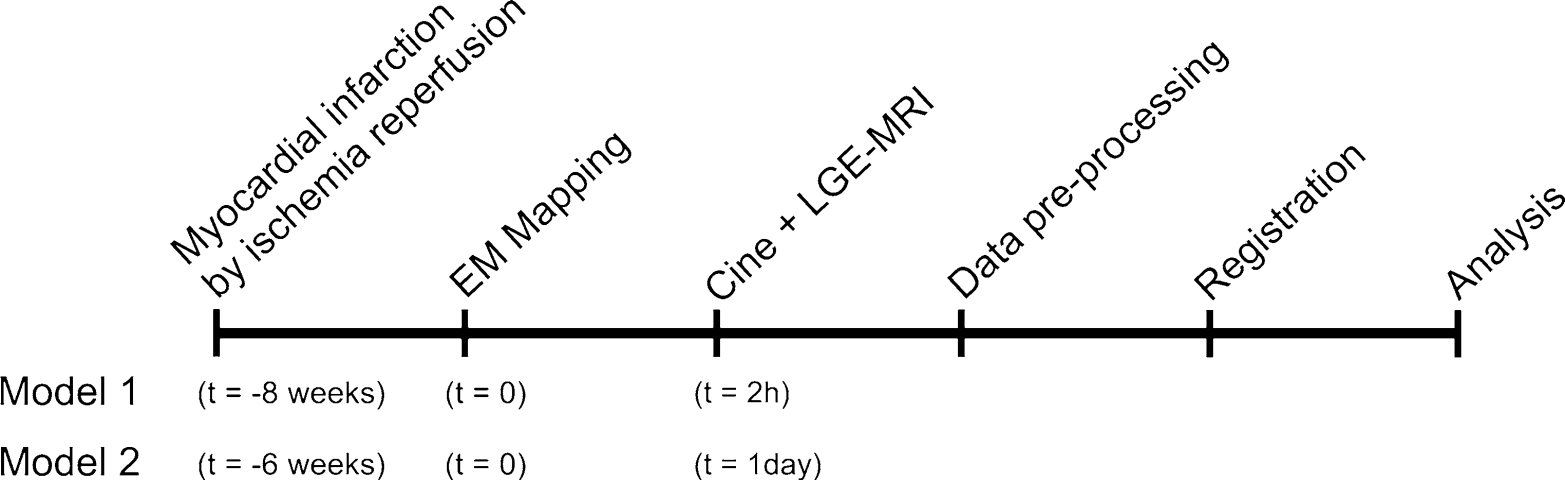
Time line of the experiments of model 1 (LCX), and model 2 (LAD)

The experiments were performed as shown in Fig. 1. Both animal models have a period between the myocardial infarction and the therapy of more than 3 weeks so that a chronic infarct can be expected [15].

### Data acquisition

For both models, the NOGA^®^ XP system (Biosense Webster, Cordis, Johnson & Johnson, USA) version 1.1.43 was used equipped with a 7 French NOGA mapping catheter (Biosense Webster, Cordis, Johnson & Johnson, Diamond Bar, USA) for the mapping procedure. The left ventricle (LV) was entered via the left carotid artery (LCX model) and the femoral artery (LAD model), and retrograde passage through the aortic valve. The catheter tip location is tracked throughout the complete cardiac cycle. Data storage is triggered on the R-wave, providing only end diastolic measurement values and points for registration. Electrocardiograms were filtered at 30–400 Hz (bipolar) and 1–240 Hz (unipolar). The EMM datasets were acquired in consideration of the criteria for good electromechanical mapping [7].

In the LCX model MRI images were acquired using a 1.5T Philips Medical systems Achieva scanner with a surface cardiac array coil. BFFE Cine scans were made to assess cardiac function, and for registration of the NOGA map on the end diastolic endocardium. The settings during the MRI acquisitions were as follows. BFFE: repetition time (TR)/echo time (ET) = 2.9 ms/1.45 ms. Flip angle = 55°, voxel size = 2.43 × 2.43 mm, field of view (FOV) = 35 × 35 cm, 144 × 144 matrix, 5 mm slice thickness, 30 phases/R to R interval, electrocardiographic gated. LGE: repetition time (TR)/echo time (ET) = 4.61 ms/1.41 ms, flip angle = 15°, voxel size = 1.36 × 1.36 mm, field of view (FOV) = 35 × 35 cm, 256 × 256 matrix, 5 mm slice thickness. In the LAD model MRI images were acquired using a 1.5T Siemens Avanto scanner with a body surface coil. BFFE: repetition time (TR)/echo time (ET) = 41.85 ms/1.18 ms. Flip angle = 68°, voxel size = 1.4 × 1.4 mm, field of view (FOV) = 35 × 25 cm, 256 × 176 matrix, 8 mm slice thickness, 25 phases/R to R interval, electrocardiographic gated. LGE: repetition time (TR)/echo time (ET) = 577 ms/1.18 ms, flip angle = 50°, voxel size = 1.4 × 1.4 mm, field of view (FOV) = 35 × 25 cm, 256 × 180 matrix, 8 mm slice thickness. Cine and LGE scans were made at the same positions with the same orientation. In both models Gadovist contrast agent (0.2 mmol/ml) was used for the LGE scans with a dose of 15 ml/kg. The scan was made 15 min after intravenous infusion of the Gadovist.

### Data pre-processing

Segmentation of the left ventricle on the short axis CINE and LGE MRI data is done in the end diastolic phase in approximately 20 slices located from apex to base. The segmentations are done automatically and checked on the long axis images using the freely available software Segment version 1.9 R2507 (http://segment.heiberg.se) [16] available for Matlab (MATLAB 2012a, The MathWorks Inc., Natick, MA, 2012). Segmentations are done to create a 3D surface mesh (cine mesh) of the LV endocardium for surface registration and projection of the acquired data. Furthermore the local wall thickening (WT) of the myocardium was assessed using the CINE images. The myocardial infarct was segmented on the LGE images using the area based semi-automatic segmentation [17]. If necessary both the LV and the infarct segmentations were manually adjusted by an experienced radiologist. Area based IT values were calculated in 80 circumferential segments of all slices using the bullseye function of Segment [17]. The IT data was projected on the CINE derived endocardial surface mesh using the TriScatteredInterp function of Matlab. The endocardial LV surface mesh is used for registration of the EMM points, image guided injection procedures, and post processing of the EMM and MRI data.

### Image registration

**Fig. 2:**
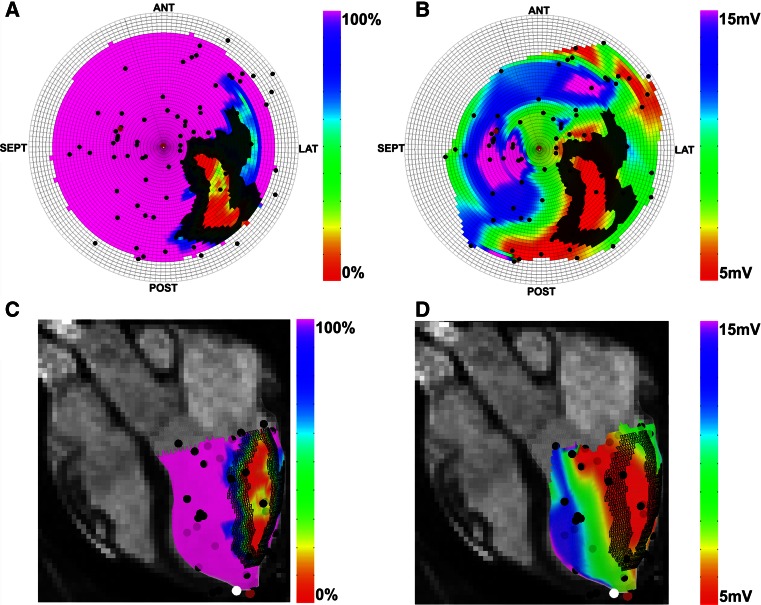
Typical images of bullseyes (**a**, **b**) and endocardial surface meshes (**c**, **d**). The *color scale* reflects the value of the infarct transmurality (**a**, **c**), Unipolar voltage (**b**, **d**). The *black dots* indicate the measurement locations of the EMM. *Brown* and *white dots* respectively represent the injection locations, and the location of the apex. The *dark hashed areas* indicate the infarct border zone as calculated by the automatic infarct border zone calculation algorithm. *ANT* anterior, *LAT* lateral, *POST* posterior, *SEPT* septum

Registration of the NOGA^®^XP and the MRI datasets was performed using a modified 3D CartBox image integration toolbox [13]. Instead of using anatomical landmarks for registration, we applied a standard rotation to align the NOGA^®^XP and the MRI coordinate systems. Hereafter we performed a rigid body translation and rotation of the NOGA^®^XP dataset based on the apex location. The remaining error after rigid body translation and rotation was caused by a slight difference of the supine positions of the animals during the EMM and MRI procedures. The registration error was minimized further by an iterative closest point (ICP) algorithm [18]. The ICP algorithm was restricted to small rotations around the apex in the sagittal, coronal, and transverse plane of respectively 10, 20 and 20° to prevent excess rotations. If necessary the registration was manually optimized by adjusting the registration interactively with six degrees of freedom: rotation and translation in the sagittal, coronal, and transverse planes. Before the registration was finalized the shape of the LV endocardial surface mesh and the EMM points was visually checked to minimize the registration error due to end diastolic volume (EDV) differences caused by the different timing between EMM and LGE-MRI acquisitions. The registration error was expressed by the mean and standard deviation of the closest distance between the EMM points and the cine mesh surface [12]. To prevent influences of EMM points located outside the cine mesh (e.g. LV outflow tract), these points were excluded for registration, and further processing. The result of the registration of a typical dataset is shown in Fig. 2.

### Analysis

The optimal thresholds of the EMM parameters to assign areas with a distinct IT were established by determining the largest area under the curve (AUC) of receiver operating characteristic (ROC) curves. ROC curves were created by automatically subdividing the IT and EMM data into 40 subsequent regions with the IT threshold ranging from ≤0 to ≤100 % with a step size of 2.5 %, and 40 subsequent regions of the EMM parameters. For UV the range was set to 0–25 mV with a step size of 0.62 mV, for BV the range was set to 0–12 mV with a step size of 0.3 mV, for LLS the range was set to −32 to 48 % with a step size of 2 % and for WT the range was set to −5 to 15 mm with a step size of 0.5 mm. To analyze the tissue that is identified by the specific UV and BV values, the infarct area was divided in 5 areas with a distinct IT: [0 %], (0−25 %], (25–50 %], (50–75 %], (75–100 %]. The classification of the IT threshold areas is shown in Table 1. To assess the capacity, and find the optimal threshold of UV and BV to distinguish areas with a distinct IT, the UV and BV values were analyzed in all IT areas. Furthermore the most distinctive threshold of WT to assess IT and LSS to assess WT were determined by respectively analyzing the WT values in areas with a distinct IT, and analyzing the LLS values in areas with a distinct WT. This analysis provides insight into the possible use of myocardial deformation assessment by MRI as an alternative technique to identify the IBZ, and assess LSS to represent myocardial deformation. To prevent misinterpretation of collagen rich low voltage areas in the valvular plane with no infarct, and therefore no LGE contrast, we excluded the basal layers of the heart from the analysis.

**Table 1:** Division of transmurality areas

Infarct area 0 % IT	All healthy tissue. IT = 0 % (includes 0 % only)
Infarct area 0–25 % IT	IT > 0 % to IT < or = 25 % (does not include 0 % and includes 25 %)
Infarct area 25–50 % IT	IT > 25 % to IT < or = 50 % (does not include 25 % and includes 50 %)
Infarct area 50–75 % IT	IT > 50 % to IT < or = 75 % (does not include 50 % and includes 75 %)
Infarct area 75–100 % IT	IT > 75 % to IT < or = 100 % (does not include 75 % and includes 100 %)

*IT* infarct transmurality

### Statistics

Statistics were performed using IBM SPSS Statistics (Version 20.0, IBM Corporation, Armonk, NY, USA). All data is presented as mean ± standard deviation (SD). The differences between the animal models were tested using a student *t* test. The differences between the points/cm^2^ of each area were calculated using the Kruskall Wallis test. We compared the EMM parameters to the IT measured by LGE-MRI per measurement point using a linear mixed model analysis to calculate the amount of IT that can be explained by the EMM parameters. For IT, the amount of residual variance (σ^2^) within the animals and the variance (intercept, *τ*) between animals were calculated (null model). Next, EMM parameters were added to the model (full model). Variance explained by the input parameter, R^2^ [19], was calculated as 100 % minus the ratio of the full and null models (Eq. 1). Hence, R^2^ represents the reduction in variance of the outcome parameter (IT) after adding input parameters (EMM; UV, BV, LLS).

1 $$R^{2} = 1 - \frac{{\sigma_{full}^{2} + \tau_{00full} }}{{\sigma_{null}^{2} + \tau_{00null} }}$$

## Results

### Animals

In all animals the myocardial infarction was in a chronic state at the time of the treatment. In the LCX model the infarct was located in the midlateral wall (3 animals). In the animals that underwent an LAD occlusion the infarct was located apicoseptally and mid-apical anterior (14 animals). All LGE-MRI datasets showed a clear hyper intense area on LGE-MRI, and cardiac function was decreased with a mean LV ejection fraction (LVEF) of 42 ± 8.7 %. The LCX and LAD models showed an LVEF of 35.7 ± 3.9 and 43.3 ± 3.9 %, respectively (*p* = 0.05). The infarct volumes of both models was not different 16.1 ± 2.2 and 15.4 ± 4.4 ml for the LCX and LAD model, respectively (*p* = 0.75). MRI data is presented in Table 2.

**Table 2:** Results of cine and late enhancement magnetic resonance imaging of 17 animals

LV end-diastolic volume (ml)	123.6 ± 32.6			
LV end-systolic volume (ml)	72.8 ± 26.4			
LV stroke volume (ml)	50.7 ± 11.5			
LV ejection fraction (%)	42.0 ± 8.7			
LV ejection fraction (%) per model (*p* = 0.05)	35.7 ± 3.9	(Model 1)	43.3 ± 9	(Model 2)
Heart rate	92.7 ± 20.7			
Myocardium volume (ml)	111 ± 15.6			
Infarct volume (ml)	15.6 ± 4.1			
Infarct volume (ml) per model (*p* = 0.75)	16.1 ± 2.2	(Model 1)	15.5 ± 4.4	(Model 2)
LV area (cm^2^)	102.7 ± 13.8			
Infarct area 0 % IT (cm^2^)	68.2 ± 12.8		(66.1 ± 6.1 %)	
Infarct area 0–25 % IT (cm^2^)	10.6 ± 3.2		(10.5 ± 3.3 %)	
Infarct area 25–50 % IT (cm^2^)	6.5 ± 3.5		(6.3 ± 3.4 %)	
Infarct area 50–75 % IT (cm^2^)	6.4 ± 2.2		(6.4 ± 2.6 %)	
Infarct area 75–100 % IT (cm^2^)	10.9 ± 7.3		(10.5 ± 6.3 %)	
Infarct areas (cm^2^) per model				
Infarct area 0 % IT (cm^2^) (*p* = 0.72)	72.6 ± 22.1	(Model 1)	67.3 ± 10.9	(Model 2)
Infarct area 0–25 % IT (cm^2^) (*p* = 0.54)	12.4 ± 4.8	(Model 1)	10.2 ± 2.9	(Model 2)
Infarct area 25–50 % IT (cm^2^) (*p* = 0.24)	10.2 ± 4.5	(Model 1)	5.7 ± 2.9	(Model 2)
Infarct area 50–75 % IT (cm^2^) (*p* = 0.88)	3.7 ± 3.7	(Model 1)	11.7 ± 6.5	(Model 2)
Infarct area 75–100 % IT (cm^2^) (*p* = 0.38)	3.8 ± 3.9	(Model 1)	12.4 ± 6.7	(Model 2)

Data are expressed as mean ± SD. The infarct area size of both models is tested using a student *t* test

*IT* infarct transmurality

### Data acquisition

The LGE-MRI datasets showed a clear myocardial infarction without microvascular obstruction as assessed by the myocardial infarct segmentation algorithm. The mean endocardial surface area in the [0 %], (0–25 %], (25–50 %], (50–75 %], (75–100 %] IT subdivisions respectively was: 68.2 ± 12.8, 10.6 ± 3.2, 6.5 ± 3.5, 6.4 ± 2.2 and 10.9 ± 7.3 cm^2^ thereby covering 66.1 ± 6.1, 10.5 ± 3.3, 6.3 ± 3.4, 6.4 ± 2.6 and 10.5 ± 6.3 % of the total LV endocardial surface area. Although it is not significant, the animal models show a marked difference in the sizes of the areas with a distinct infarct transmurality. The infarct of the LCX model has a larger portion in the 0–50 % IT region whereas the LAD model has a larger portion in the 50–100 % IT region. MRI data is presented in Table 2. The mean number of EMM points in the EMM acquisitions was 152.3 ± 55. All EMM parameters are displayed in Table 3.

**Table 3:** Three dimensional electromechanical mapping and image registration results of 17 animals

Electromechanical mapping points				
Total number of EMM points	2867			
EMM points used for registration	2590			
Points per animal	152.3 ± 55			
Registration error (mm)	3.01 ± 1.94			
Registration error (mm) per model (*p* = 0.32)	3.22 ± 1.87	(Model 1)	3.0 ± 1.82	(Model 2)
Points used for projection and analysis				
Total number of EMM points	1581			
Points per animal	93 ± 27.2			
Points per infarct transmurality area used for projection and analysis				
0 % IT	50.2 ± 18.4	0.7 ± 0.3 points/cm^2^		
0–25 % IT	9.7 ± 6.2	0.9 ± 0.5 points/cm^2^		
25–50 % IT	8.0 ± 4.3	1.4 ± 0.7 points/cm^2^		(*p* < 0.05)
50–75 % IT	6.3 ± 5.6	0.9 ± 0.6 points/cm^2^		
75–100 % IT	18.7 ± 14.2	1.5 ± 0.8 points/cm^2^		

Data are expressed as mean ± SD. The registration error of both models is tested using a student *t* test. The differences between the points/cm^2^ of each area are calculated using the Kruskall Wallis test

*IT* infarct transmurality

### Image registration

The registration data is summarized in Table 3. The total number of EMM points in the 17 datasets was 2867. After exclusion of points outside the mesh, the total number of points that are used for registration and projection of EMM parameters on the endocardial surface mesh was 2590. The EMM points acquired during the mapping procedure were aimed to be as homogeneously distributed over the endocardial surface as possible to assure optimal registration of the EMM and MRI cine mesh. The resulting mean registration error of all datasets was 3.01 ± 1.94 mm and the mean registration error of the LCX and LAD models were 3.22 ± 1.87 and 3,0 ± 1,82 (*p* = 0.32), respectively. The EMM point density was significantly higher in the regions with 25–50 and 75–100 % IT (*p* < 0.05).

### Analysis

**Fig. 3:**
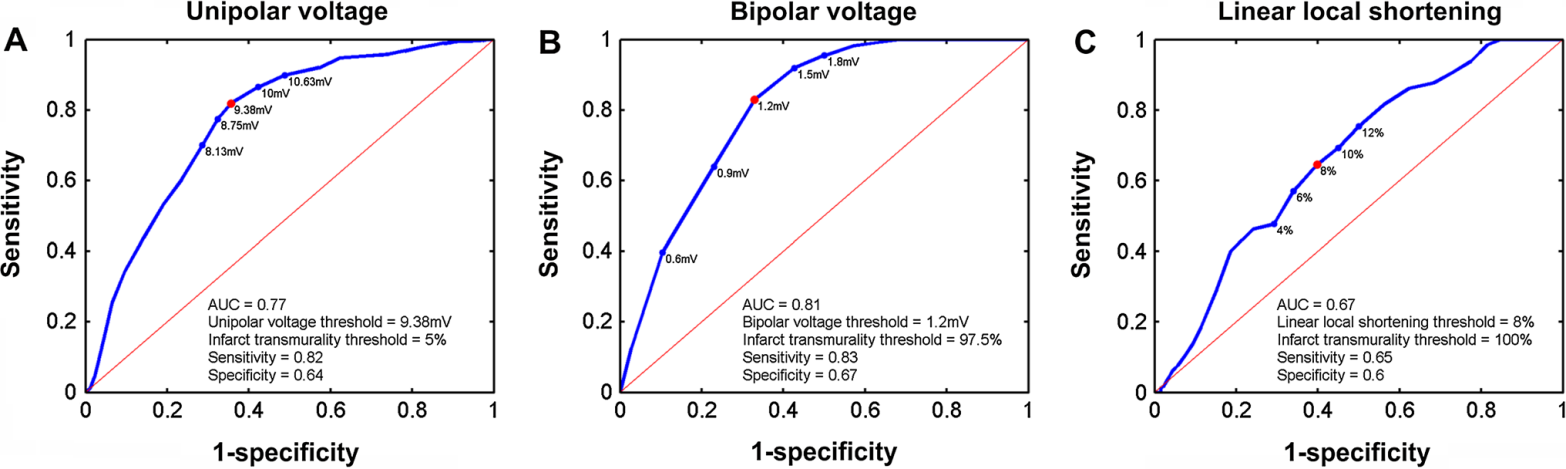
ROC curves for the unipolar voltage (**a**), bipolar voltage (**b**), linear local shortening (**c**) with the highest AUC. The corresponding thresholds of the respective parameters, and the infarct transmurality are annotated in the figures. The *red dot* marks the highest sensitivity and specificity

**Fig. 4:**
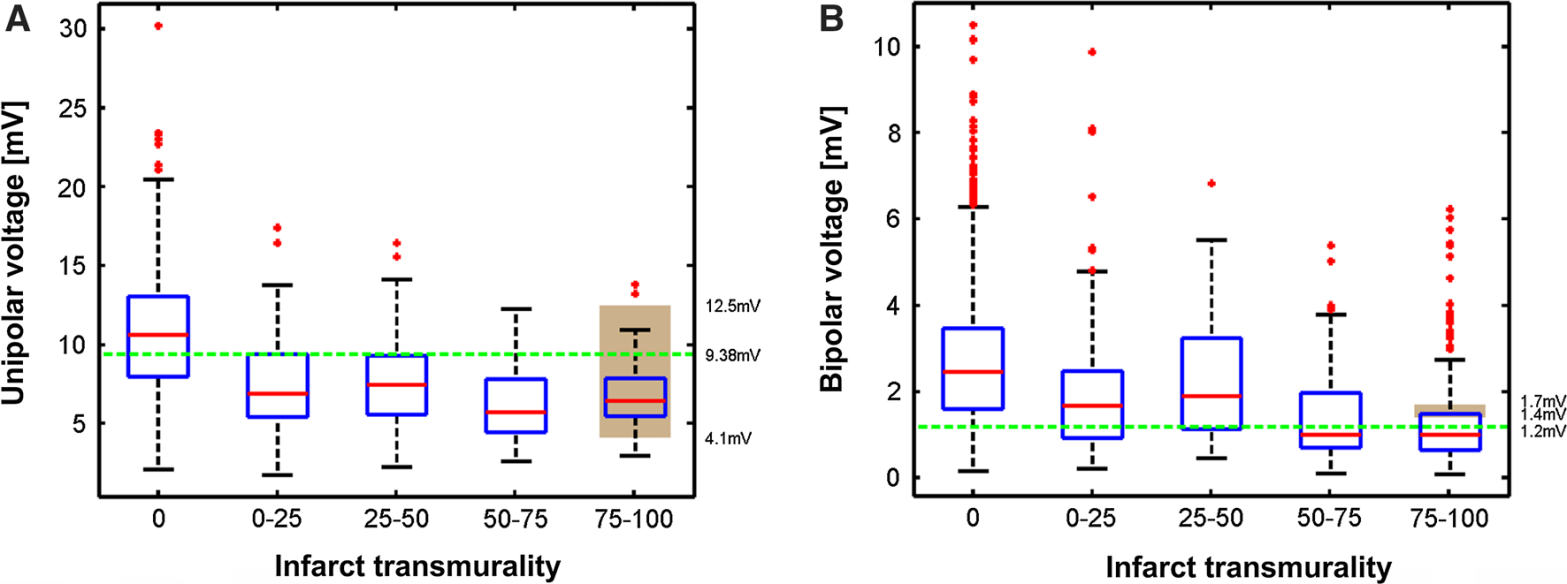
Box plots of the distribution of the unipolar voltage (**a**), bipolar voltage (**b**) values in the areas with a distinct infarct transmurality. *Boxes* represent the 25–75 % percentiles and the *red line* in the boxes represents the median value. *Red dots* are outliers (>3 × SD from the mean value). The *green line* represents the value with the highest AUC as shown in Fig. 1. The *grey area* represents the range of the threshold values reported in literature to distinguish core infarct. Numbers on the right annotate the range of the threshold values and the value of the *green line*

**Fig. 5:**
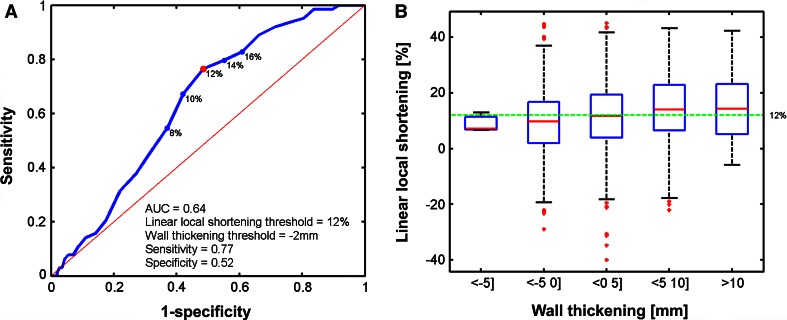
ROC curve for linear local shortening with the highest AUC (**a**). The corresponding thresholds for wall thickening and linear local shortening are annotated in the figure. *Box plots* of the distribution of the linear local shortening values compared to wall thickening (**b**). *Boxes* represent the 25–75 % percentiles and the *red line* in the boxes represents the median value. *Red dots* are outliers (>3 × SD from the mean value). The *green line* represents the value with the highest AUC

**Fig. 6:**
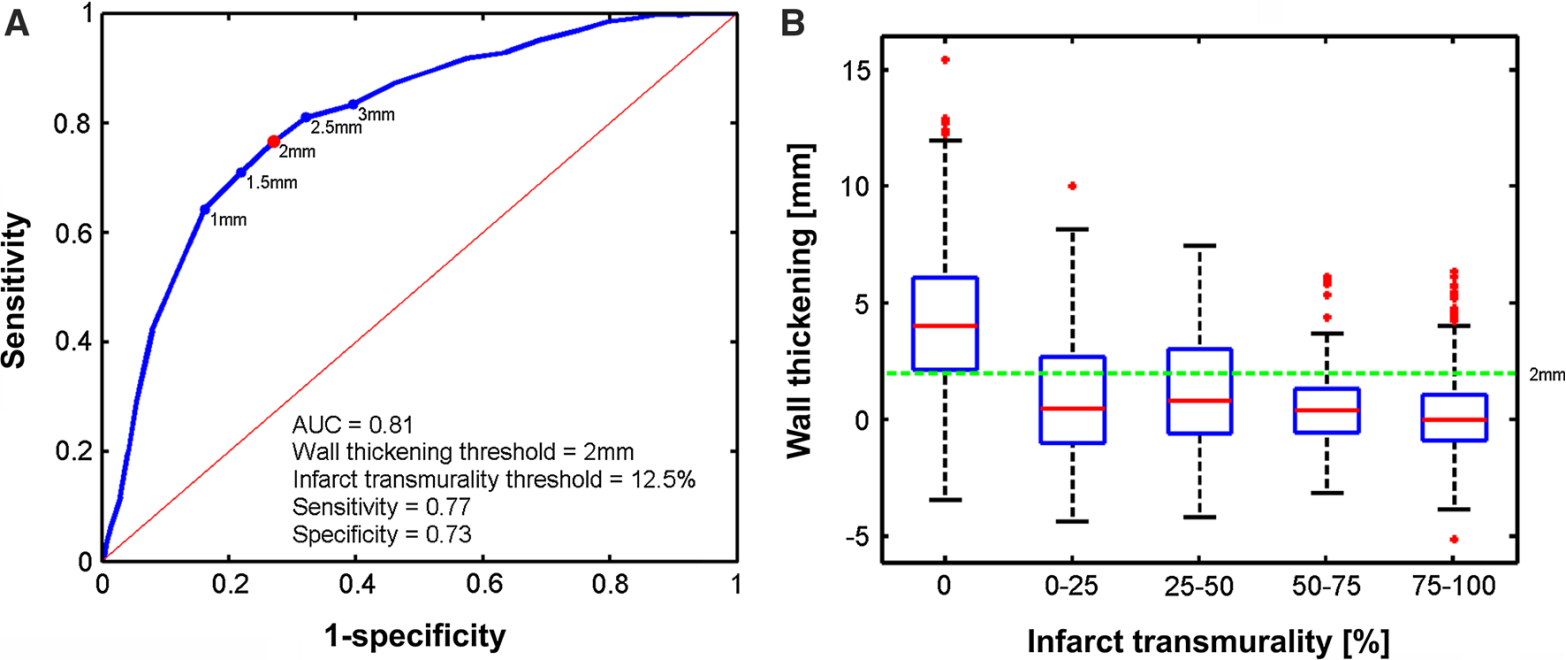
ROC curve for wall thickening with the highest AUC (**a**). The corresponding thresholds for wall thickening and infarct transmurality are annotated in the figure. *Box* plots of the distribution of the wall thickening values compared to infarct transmurality. *Boxes* represent the 25–75 % percentiles and the *red line* in the *boxes* represents the median value. *Red dots* are outliers (>3 × SD from the mean value). The *green line* represents the value with the highest AUC

ROC curves of the EMM parameters are shown in Fig. 3. For each EMM parameter the ROC with the highest AUC to detect areas with IT ≥0 % are shown (Fig. 4). UV best determines IT ≥5 % (AUC = 0.77) when using a threshold of ≤9.38 mV with a sensitivity of 0.82 and specificity of 0.64. The mixed model analysis showed that UV explained 15 % of IT (Table 4). BV best determines IT ≥97.5 % (AUC = 0.81) when using a threshold of ≤1.2 mV with a sensitivity of 0.83 and specificity of 0.67. From the mixed model analysis it is appreciated that BV explains 10 % of IT (Table 4). The lowest AUC is found for LLS (AUC = 0.67) to distinguish IT ≥100 % by LLS ≤8 % (sensitivity = 0.65, specificity = 0.6). Correspondingly the mixed model analysis also shows that only 3 % of IT is explained by LLS (Table 4). Furthermore we have assessed the relations between LLS and WT, and between IT and WT. LLS most accurately discerns regions with IT of 100 % as was shown in Fig. 3c and is poorly related to local WT (Fig. 5). The ROC curve in Fig. 5a shows that LLS <12 % distinguishes WT ≥2 mm with sensitivity = 0.77 and specificity = 0.52. Nevertheless an analysis of the WT and IT both measured by MRI shows that WT is related to local IT. In Fig. 6 it is shown that WT determines IT ≥12.5 % (AUC = 0.81) when using a threshold of ≤2 mm, with a sensitivity of 0.77 and specificity of 0.73.

**Table 4:** Comparison of EMM parameters with infarct transmurality

Parameter	R^2^ (%)
UV	15
BV	10
LLS	3

R^2^ explained variance of myocardial fibrosis by the regarding parameter

## Discussion

In this study we have retrospectively applied the 3D CartBox image integration toolbox to a dataset of 17 pigs with a chronic MI. For the first time we have used 3D CartBox in a larger group resulting in a mean surface registration error of 3.01 ± 1.94 mm. In contrast to earlier studies using a manual segmental comparison [7, 9], this technique enabled an accurate in depth analysis of the electromechanical properties of infarct areas. The six main findings of this study are: (1) The use of 3D CartBox is feasible. (2) UV and BV do distinguish infarcted and healthy tissue, but have less sensitivity to clearly identify regions with a distinct IT value based on LGE-MRI. (3) LLS and WT are differently affected by IT. (4) WT is affected in the infarct areas, but does not reflect areas with different IT values. (5) Integrated EMM and LGE-MRI images have the potential to guide intramyocardial injections more accurately than the on-table EMM and off-table LGE-MRI alone. (6) The applied EMM-LGE-MRI fusion software can be used for *clinical applications of real*-*time* image guided intramyocardial injections in the future.

### Animals

This study was conducted in a multicenter fashion using two different animal models. However not significant, in the LCX model the sizes of the 100 % transmurally infarcted regions were smaller (Table 2). One animal with an LCX infarct had no 75–100 % IT area 8 weeks after MI. Most likely this was caused by lower ischemia time of 75 min which causes less irreversible cardiomyocyte cell death. In the 90 min LAD occlusion model all pigs had a transmural infarction 6 weeks after MI. No microvascular obstructions were detected in the pigs as could be expected based on literature [20]. Because clear infarcts with different IT regions were established in both animal models, all datasets qualified to be used for further analysis.

### Data acquisition

Although data of the two centers were recorded using different imaging equipment, data acquisition protocols were identical, and therefore image registration and analysis could be performed using the 3D CartBox toolbox using standard image formats for MRI (DICOM) and NOGA^®^XP (SQL database).

### Image registration

Due to a low spatial resolution of the EMM, the EMM surface meshes never reflect the exact LV surface and therefore a registration error will always occur. In both models the EMM and MRI acquisitions were performed within 2 h (LCX) and 1 day (LAD). A longer time between EMM and LGE-MRI acquisitions increases the chance of a difference in cardiac load/filling during the EMM and MRI procedures, which can cause an increase of the registration error. Since we did not observe a significant difference between the registration errors of the different models, we believe that the load/filling difference during EMM and LGE-MRI acquisitions was minimal. In this study we have used the standard rotation to align the NOGA^®^XP and MRI coordinate systems, instead of initial registration based on anatomical landmarks [13]. Since the pigs were in supine position during both the EMM and MRI procedures the orientation of the animal in during the EMM and MRI procedures could be standardized to minimize the remaining registration error. To prevent erroneous rotations by the subsequent ICP and manual optimization steps of the registration, we have secured that rotations during the manual optimization steps did not exceed 5°. The resulting registration error is 3.01 ± 1.94 mm, which is clinically acceptable and even slightly better than the values reported in studies where CARTO^®^ was used: 3.83 ± 0.57 mm [12] or 4.3 ± 3.2 mm [11]. The SD of the registration error might be affected by the regularity of the heart rate during the EMM procedures resulting in end diastolic LV surface alterations in the EMM acquisition, or respiratory induced motion of the heart. The NOGA^®^XP system does not compensate for this. From Table 3 it can be observed that the EMM points are not homogeneously distributed over the areas with a different IT. The highest point density is found in the area with 75–100 % IT. This most likely is caused by the targeted search for the infarct area with the EMM catheter during the mapping procedure or the preference of the cathetertip for aneurysms located in the core infarct.

### Analysis

As already visually noticed [10, 12], infarcted areas measured by LGE-MRI do not always overlap with infarct areas identified by EMM. In our data we have found that EMM has the highest sensitivity and specificity to identify infarct transmuralities of ≥5 and ≥97.5 % by electric signals UV <9.38 mV and BV <1.2 mV respectively as is shown in Fig. 3. The different sensitivity for different areas most likely is caused by the detection of far field depolarization signals by a single electrode (UV), which are filtered out by the subtraction of two electrode signals that are positioned within 2 mm on the catheter (BV). Infarcted tissue in the vicinity of the measurement decreases UV in a larger area whereas BV is only affected by the local tissue. UV therefore performs best to identify areas that are located more at the borders of the infarct, whereas BV is more eligible to identify areas that are located at the infarct core. The linear mixed model analysis presented in Table 4 showed poor overlap between the EMM parameters and the IT based on LGE-MRI. The large range of the EMM parameters in each area with a distinct IT (Fig. 4) emphasizes that the EMM parameters do not accurately reflect IT. This variance might partly be induced by the registration error or the difference between infarct assessment by EMM and LGE-MRI. Since the administration-scanning procedure of the LGE-MRI scan is performed accurately and according to clinical standard, it is expected that the LGE-MRI scans are correct and therefor used as a reference in this study. Potential error sources are the measurements of low depolarization values on healthy tissue due to limited wall contact or measurement of high depolarization values on infarcted tissue caused by far field signals or the presence of local patches of healthy tissue. Since irregular points in the EMM dataset are usually re-examined by the operator, this effect is expected to be a small source of error. On a cellular level depressed electrical activity in the myocardium adjacent to the infarct might play a role [15, 21]. The inhomogeneous distribution of wall stress during the remodeling process causes the myocardium to enter the transient phase of collagen deposition. In the transient phase uptake of gadolinium and the decreased electrical function might be out of phase. Unfortunately studies using a comparable registration algorithm in the field of electrophysiology either did not calculate the ROC curves [12], or did not perform a detailed analysis of the areas with a different IT [10, 11]. The wide range of threshold values represented in literature [7] to identify core infarct as shown by the gray area in Fig. 4 results from the use of different definitions of viability/perfusion/transmurality and the use of multiple absolute (PET, echocardiography, histology) and relative (SPECT) techniques to identify this, in combination with a manual segmental comparison to EMM data [9]. To be able to target the hibernating tissue that is mechanically depressed and electrically active, the LLS parameter was introduced to measure local relative cathetertip movements caused by cardiac deformation. Areas with severely depressed movements (LLS) and a maintained UV or BV should present the hibernating tissue in the IBZ. Strikingly Fig. 5 illustrates that there is no relation between LLS and the local WT [9]. This is most likely caused by the definition of LLS being: ‘the change in distance between an index point and all its recorded surrounding points from late diastole to maximum systole’ [22]. A weighing is applied to emphasize points within a distance of 8–15 mm [22]. Consequently for a point in an area with small number of points or points moving due to tethering of the tissue, LLS is based on points located more distantly, or based on a false perception, causing erroneous LLS values. To correctly assess cardiac deformation by LLS a higher point density is necessary. In summary, the identification of the IBZ by EMM can be improved by using fusion software to combine EMM and LGE-MRI. Strategies to more optimally identify the IBZ based on LGE-MRI might be helpful to increase the effects of cardiac regenerative therapy.

## Clinical implications

Advanced ROC analysis revealed cutoff values of UV <9.38 mV and BV <1.2 mV to respectively distinguish areas with ≥5 and ≥97.5 % IT. For clinical perspective we have shown that EMM is an adequate tool to distinguish transmurally infarcted myocardium from normal myocardium. For more detailed quantification of transmurality, we propose to incorporate LGE-MRI data. This may be of interest in view of targeting the exact border zone [23], The 3D CartBox toolbox for image registration can be used to further specify the most optimal injection location for cardiac regenerative therapy based on LGE-MRI.

## Limitations

For both BV and LLS we found that the acquisition of more EMM measurement points would probably have given more insight into the full potential of the BV and LLS parameters. Furthermore the retrospective design of this study and lack of histological data made it impossible to perform a detailed comparison between EMM, LGE-MRI, and histology. In prior studies using coarse registration techniques a good agreement was found between EMM and histology [24] and between LGE-MRI and histology [25–27]. Such a study is necessary to assess the accuracy of LGE-MRI guided injections and will be performed in a 3D fashion in the near future. During the mapping procedure small respiratory induced excursions of the catheter tip were observed. Since the NOGA^®^XP system does not compensate for respiratory induced motion of the cathetertip, this most likely affected the registration accuracy. Compensation for respiratory motion might be beneficial for future applications.

## Conclusion

We are the first to perform robust 3D registration and an in depth comparison of the NOGA^®^XP EMM parameters and IT measured by LGE-MRI to determine the infarct identification characteristics of EMM. We found that the 3D CartBox image registration toolbox enables multimodality infarct identification and can be used for further specification of the most optimal injection locations for cardiac regenerative therapy.
